# Sequencing of glycosaminoglycans with potential to interrogate sequence-specific interactions

**DOI:** 10.1038/s41598-017-15009-0

**Published:** 2017-11-01

**Authors:** Toin H. van Kuppevelt, Arie Oosterhof, Elly M. M. Versteeg, Emina Podhumljak, Els M. A. van de Westerlo, Willeke F. Daamen

**Affiliations:** Department of Biochemistry, Radboud Institute for Molecular Life Sciences, Radboud university medical center, PO Box 9101, 6500 HB Nijmegen, The Netherlands

## Abstract

Technologies to sequence nucleic acids/proteins are widely available, but straightforward methodologies to sequence complex polysaccharides are lacking. We here put forward a strategy to sequence glycosaminoglycans, long linear polysaccharides involved in many biochemical processes. The method is based on the covalent immobilization and (immuno)chemical characterization of only those size-separated saccharides that harbor the original reducing end of the full-length chain. Using this methodology, the saccharide sequence of the chondroitin sulfate chain of the proteoglycan bikunin was determined. The method can be performed in any standard biochemical lab and opens studies to the interaction of complex saccharide sequences with other biomolecules.

## Introduction

In addition to nucleic acids and proteins, glycosaminoglycans have been indicated as a class of information-dense biomolecules. Glycosaminoglycans, which are long, linear, anionic polysaccharides generally linked to a core protein (proteoglycans), are present at the cell surface and in the extracellular matrix and are involved in many biological processes^1–3^. They are composed of up to ~100 disaccharides and their biosynthesis allows for a large structural variability including differential sulfation, acetylation and epimerization. Analogous to nucleic acids and proteins, glycosaminoglycans have polarity, *i.e*. a beginning (the reducing end) and an end (the non-reducing end). Whereas the existence of a defined disaccharide sequence is still debated, the presence of domain structures has been amply indicated including their role in the binding and modulation of proteins like growth factors and cytokines.

Sequencing of glycosaminoglycans has proven extremely difficult. Few attempts have been reported, including the use of exoenzymes on defined oligosaccharides^4,5^, the use of radioactive end-labeling^6,7^, and the application of sophisticated mass spectrometry^8,9^. These methods generally reveal information on oligosaccharide stretches of glycosaminoglycans, or have a high level of complexity. None of the methods allows for the study of saccharide sequence-specific interaction with other biomolecules like proteins or with cells.

**Figure 1:**
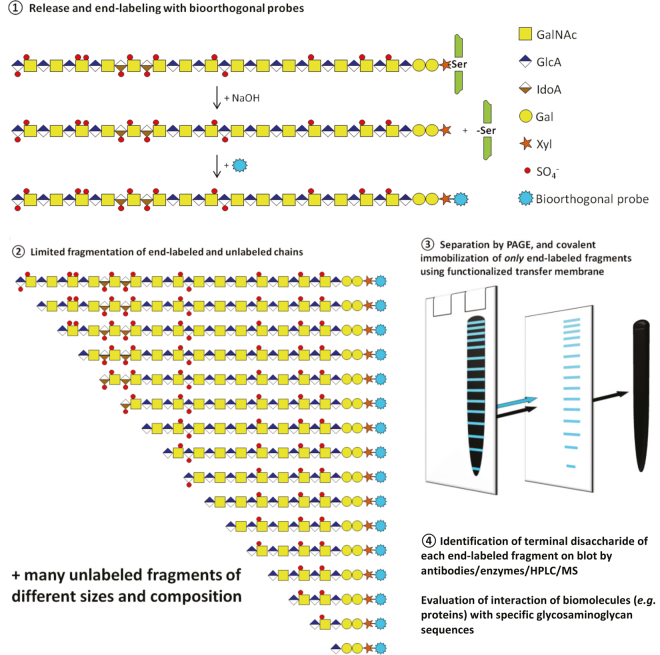
Outline of strategy to sequence glycosaminoglycans. Glycosaminoglycans are liberated from proteoglycans using NaOH (ß-elimination) and labeled with a bioorthogonal group at the resulting reducing end (step ①). Next, glycosaminoglycans are fragmented (*e.g*. by enzymes) and size-separated by PAGE (step ②). Labeled fragments, *i.e*. those with the original reducing end, are then separated from unlabeled fragments (indicated as a black smear) by blotting to paper functionalized with the biorthogonal reaction partner (“click chemistry”, step ③). Only labeled fragments will be retained by the paper, the unlabeled ones passing through. This results in a set of size-separated fragments, all with the original reducing end and separated from each other by one terminal disaccharide. By establishing the nature of the terminal (non-reducing) disaccharide in each band (*e.g*. by specific antibodies, HPLC, MS, step ④), the sequence of the glycosaminoglycan can be read. Glycosaminoglycan fragments are freely accessible allowing sequence-specific interrogation by biomolecules like proteins and by cells. Since fragments are covalently attached to the blotting paper, chemical treatments can be performed *in situ*.

Analysis of glycosaminoglycans generally comprises enzymatic or chemical fragmentation resulting in a large number of saccharides originating from all parts of the full-length polysaccharide. This impedes sequence analysis. To tackle this problem, we developed ways to specifically immobilize and identify only those saccharide fragments that harbor the original reducing end of the glycosaminoglycan (see Fig. 1 for strategy). To this end, we used bioorthogonal reaction pairs (“click chemistry”), labeling the reducing end of glycosaminoglycans with one reaction partner, and transferring them to blot paper functionalized with the other reaction partner.

## Results and Discussion

### Labeling of the reducing site of saccharides with bioorthogonal probes

**Figure 2:**
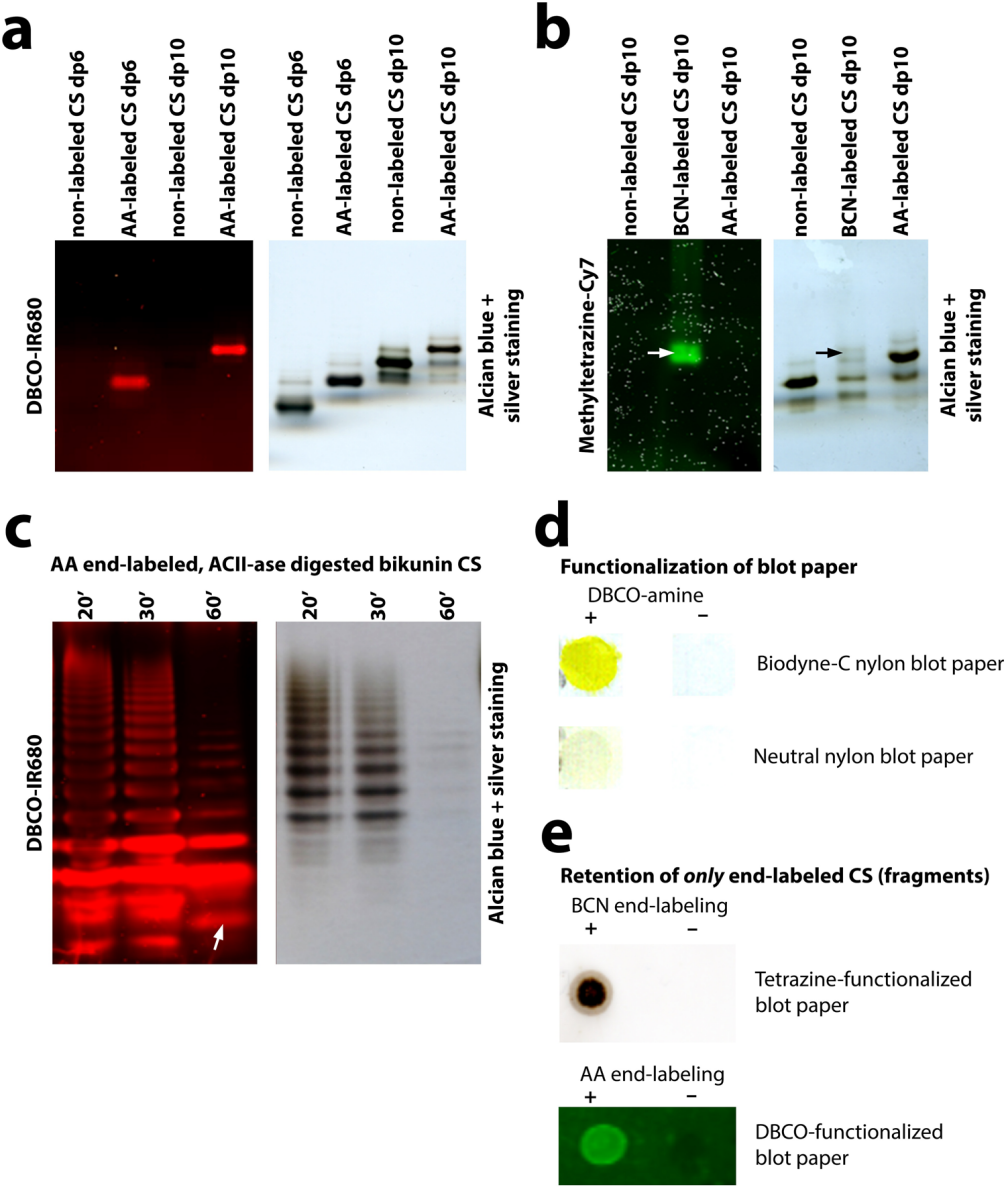
End-labeling, detection and specific immobilization of chondroitin sulfates using bioorthogonal probes. (**a)** End-labeling of chondroitin sulfate hexa- and decasaccharides (dp6 and dp10) with azidoaniline (AA) and detection by DBCO-IR Dye680RD^®^ or by Alcian Blue/silver staining. Both stainings are of the same gel. (**b**) End-labeling of chondroitin sulfate decasaccharide (dp10) with BCN-PEG_3_-amine or with azidoaniline (AA), and detection by Cy7-methyltetrazine or Alcian Blue/silver staining. Note moderate labeling (black arrow), but good staining (white arrow) for BCN-PEG_3_-amine. The AA-labeled decasaccharide is not detected because Cy7-methyltetrazine is not a click partner of AA. Both stainings are of the same gel. (**c**) Detection of chondroitinase AC-II derived fragments from full-length, AA end-labeled chondroitin sulfate derived from the proteoglycan bikunin, and detected by DBCO-IR Dye680RD^®^ or Alcian Blue/silver staining. Enzyme incubation times were 20, 30 and 60 min. About 15 bands are visible. Note that fast migrating bands are not stained with Alcian Blue/silver. Both stainings are of the same gel. The arrow indicates an additional band observed after prolonged enzyme digestion (see also Supporting Information Fig. 2). (**d**) Functionalization of blot paper with the bioorthogonal probe DBCO using EDC/NHS coupling. DBCO was visualized by dinitrophenyl-azide (yellow). Note superior functionalization of BiodyneC nylon blot paper. (**e**) Immobilization of only end-labeled chondroitin sulfate (derived from bikunin). Upper part: BCN end-labeled chondroitin sulfate on tetrazine-functionalized blot paper: silver staining. Lower part: AA end-labeled chondroitin sulfate fragments on DBCO-functionalized blot paper. Detection by antibody 1B5.

As a first step, we evaluated bioorthogonal probes used in strain-promoted azide-alkyne cycloadditions and in alkene/alkyne-tetrazine inverse electron demand Diels-Alder reactions for labeling of the reducing end of saccharides by reductive amination (Table 1). Oligosaccharides were used as model compounds and labeling was evaluated by a shift in migration after polyacrylamide gel electrophoresis (Fig. 2a,b).

**Table 1:** Labeling efficiency of various bioorthogonal probes with chondroitin sulfate decasaccharide.

Probe	Labeling efficiency
azidoaniline	+++
azido -PEG_3_-oxyamine	++
tetrazine-oxyamine	++/+++*
tetrazine-amine	+
dibenzylcyclooctyne-amine	+
bicyclononyne-PEG_3_-amine (endo)	+
*trans*-cyclooctene -PEG_3_-amine	+
*trans*-cyclooctene-amine	+

+++: nearly complete; ++: good (about half or more); +: moderate.

*labeling is better without reduction by NaCNBH_3_.

Labeling was performed using 0.1 M probe in 100 mM NaAc/150 mM NaCl, pH 5.4. After 15 min at ambient temperature, an equal volume of 1 M NaCNBH_3_ was added and incubation proceeded for another 4½ h at 45 °C.

Bioorthogonal probes functionalized with arylamines (azidoaniline) gave nearly complete labeling, whereas probes with primary alkyl-amines (*e.g*. dibenzylcyclooctyne (DBCO)-amine and bicyclononyne (BCN)-PEG_3_-amine) reacted only moderately. Oxyamine-containing compounds (*e.g*. azide-PEG_3_-oxyamine and tetrazine-oxyamine) labeled well (>50%), the reduction by sodium cyanoborohydride not being necessary. This may be advantageously since reduction may inactivate probes as was seen for tetrazine-based compounds. End-labeled saccharides could be specifically visualized in gel by incubation with their click partner conjugated to an IR dye, whereas non-labeled saccharides did not stain (Fig. 2a,b). Detection sensitivity matched that of silver staining (~20–30 ng) with the major advantage that small saccharides, not detected by silver staining, were stained (*e.g*. Figure 2c).

### Fragmentation and in-gel visualization of end-labeled chondroitin sulfate

To fragment glycosaminoglycans into a nested set of saccharides differing only one disaccharide from each other, we used chondroitinases and separation by polyacrylamide gel electrophoresis^10,11^. To evaluate the sequence procedure (Fig. 1) we used the commercially available proteoglycan bikunin which has a single chondroitin sulfate chain of which sequence information is known^8,12^. Bikunin has an apparent molecular mass of about 30–37 kDa, the chondroitin sulfate chain consisting of about two thirds non-sulfated disaccharides (0S) and one third 4-sulfated (4S) disaccharides (Supplementary Information Fig. 1). The full-length chondroitin sulfate was liberated from the core protein by NaOH treatment (alkaline ß-elimination) (Supplementary Information Fig. 1b), and end-labeled with azidoaniline (note that labeling is on the first sugar (xylose) of the tetrasaccharide linkage region which attaches the glycosaminoglycan chain to the core protein). After partial digestion using chondroitinase AC-II, labeled fragments were detected in gel using the bioorthogonal partner DBCO conjugated to IR Dye680RD^®^ revealing about 15 clearly separated bands (Fig. 2c).

### Sequencing of chondroitin sulfate

**Figure 3:**
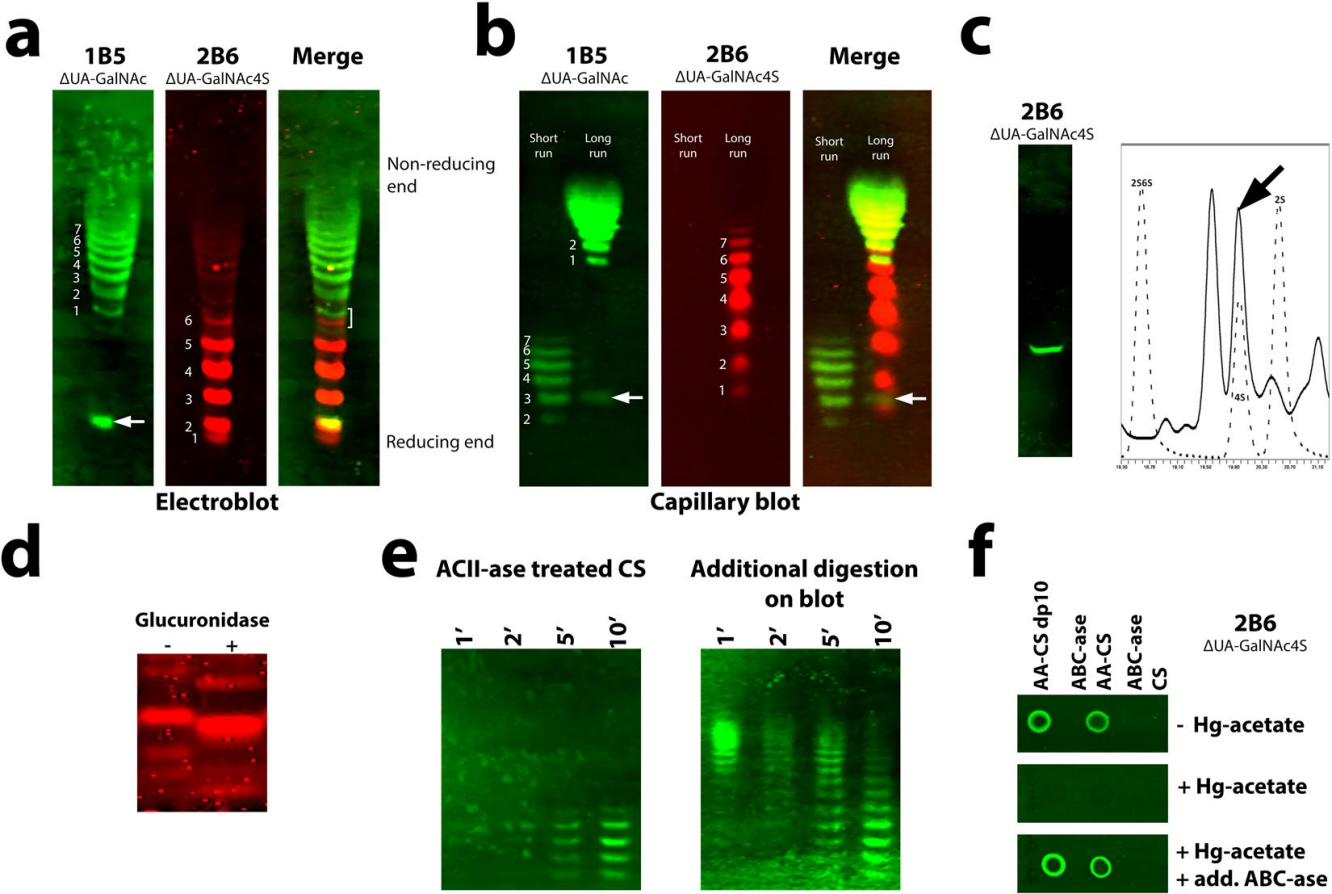
Sequencing of chondroitin sulfate from the proteoglycan bikunin and (bio)availability of sequence-defined fragments. (**a**) BCN end-labeled chondroitin sulfate digested with chondroitinase AC-II, separated by PAGE and blotted to tetrazine-functionalized paper followed by incubation with the antibody 1B5. From the staining pattern the sequence can be read (arrow: OS disaccharide close to reducing end; square bracket: the transition zone). (**b**) Same as (**a**) but azidoaniline end-labeling and DBCO-functionalized blot paper. 1B5 and 2B6 stainings are on different lanes. Long and short run: electrophoresis for 5.5 and 3.5 h, respectively. (**c**) Identification of the first disaccharide. BCN-labeled chondroitin sulfate digested with chondroitinase ABC, subjected to PAGE, and blotted to tetrazine-functionalized blot paper followed by staining with antibody 2B6. A non-stained fragment was cut out of the blot paper and digested with chondroitinase AC-II releasing the first disaccharide which was identified as ∆UA-GalNAc4S by RP-HPLC (right picture, see arrow). (**d**) Identification of uronic acid moiety. Azidoaniline end-labeled chondroitin sulfate digested with hyaluronidase with or without exo-glucuronidase. The shift in migration identifies the uronic acid as glucuronic acid. (**e,f**) *In situ* (bio)availability of sequence-defined chondroitin sulfate fragments. (**e**) BCN end-labeled chondroitin sulfate was fragmented as under (**a**) and visualized using antibody 2B6. Non-staining fragments became apparent after additional on-blot digestion with chondroitinase ABC + AC, creating new antibody- 2B6 positive bands (see *e.g*. lanes labeled 5′). (**f**) Chemical availability of chondroitin sulfate fragments. Azidoaniline (AA) labeled chondroitin sulfate A and decasaccharides were immobilized on DBCO-functionalized blot paper and treated without (−, upper part) or with ( + , middle part) Hg-acetate to remove the terminal unsaturated uronic acid residue resulting in a loss of antibody 2B6 staining. Staining is restored after additional treatment with chondroitinase ABC (lower part).

After separation of the chondroitin sulfate fragment using PAGE, the end-labeled fragments are still mixed with an abundancy of unlabeled fragments. To identify their chemical nature –and hence to deduce a sequence– labeled fragments have to be isolated. For this, we functionalized blotting paper with the bioorthogonal reaction partner to allow immobilization of only end-labeled fragments, and expose them in a way allowing (immuno)chemical characterization. Amine-containing bioorthogonal probes were covalently immobilized to blot paper rich in free carboxylic groups using carbodiimide (EDC/NHS) crosslinking chemistry. We selected nylon-based BiodyneC blot paper as it contains many carboxylic groups which will also prevent non-labeled glycosaminoglycans from binding. This approach was successful (Fig. 2d,e) and applicable for all the amine-containing probes tested (*e.g*. tetrazine-amine, DBCO-amine). Having optimized the end-labeling of glycosaminoglycans with one bioorthogonal partner and blotting paper with the other, we next selected two reactions pairs for their effectivity to immobilize end-labeled glycosaminoglycan fragments on blot paper. The combination of azidoaniline-labeled fragments and DBCO functionalized blot paper was selected because of the high efficiency of labeling with azidoaniline and its small size. The combination BCN-tetrazine was chosen because of the high reaction rate constant^13,14^, allowing electroblotting. Chondroitin sulfate from bikunin was end-labeled and digested with chondroitinase AC-II resulting in fragments with a terminal unsaturated (∆) disaccharide. Fragments were separated on gel and blotted to functionalized paper. Since HPLC (Supplementary Information Fig. 1c) demonstrated that only two types of disaccharides were present (a uronic acid (UA) with either a 4-sulfated or a non (0) sulfated N-acetyl galactosamine (∆UA-GalNAc4S, ∆UA-GalNAc, respectively)), blot paper was incubated with antibodies specific for these terminal (unsaturated) disaccharides (antibody 2B6 and 1B5, respectively)^15^. Seven bands reactive with antibody 2B6 were identified at the reducing end of the chain, whereas at least 7 bands were observed towards the non-reducing end (Fig. 3a,b), indicating a sequence of a nested set of 4-O sulfated N-acetylated disaccharides at the reducing side, and a nested set of non-sulfated N-acetylated disaccharides at the non-reducing side. To identify the first disaccharide of the chondroitin sulfate chain proper (*i.e*. the first disaccharide after the linkage region), extensive digestion using chondroitinase ABC was performed resulting in the linker region + 1 disaccharide^16^ (Fig. 3c). One single 2B6 positive band was observed indicating it to be ∆UA-GalNAc4S. This was confirmed by HPLC disaccharide analysis of an identical –but unstained– band that was cut out of the blot paper and digested by the enzyme chondroitinase AC-II which cleaves off the last disaccharide before the linkage region. The disaccharide was identified as ∆UA-GalNAc4S (Fig. 3c). In the sequence deduced, there are some notable aspects. Close to the reducing end a non-sulfated disaccharide was observed located between band 1 and 2 in case of azidoaniline labeling, and close to band 2 in case of BCN labeling (Fig. 3a,b, arrows). In addition, a fragment with both ∆UA-GalNAc4S and ∆UA-GalNAc at its terminal side was observed (Fig. 3a, square bracket), indicating the transition zone between the 4- and non-sulfated clusters. When more material was blotted (Fig. 3b), two additional minor 2B6 positive bands were observed above this band (band no. 7 and a minor band above that, Fig. 3b) indicative for two additional disaccharides present at the end of the transition zone.

Having established the sequence of the N-acetylgalactosamine component, we next probed the nature of the uronic acid component of the disaccharides. HPLC analysis (Supplementary Information Fig. 1c) showed this to be either glucuronic acid or its epimer iduronic acid. Extensive digestion with chondroitinase AC-II, specific for glucuronic acid containing chondroitin sulfate, resulted in complete degradation of chondroitin sulfate. In addition, fragmentation of chondroitin sulfate by testicular hyaluronidase followed by treatment with exo-ß-glucuronidase resulted in a shift in migration of fragments most obvious for the smaller fragments (Fig. 3d). This indicates glucuronic acid as the uronic acid moiety. Combined these results indicate the sequence of the chondroitin sulfate of bikunin to be: – (GlcA-GalNAc)_5_ − (GlcA-GalNAc/GlcA-GalNAc4S)_3_^#^ − (GlcA-GalNAc4S)_4_ − GlcA-GalNAc* − GlcA-GalNAc4S − linkage region (^#^: transition zone with increasingly less 4S towards the non-reducing end, *: position not exactly known).

The sequence strategy described here relies upon the separation of fragments by size. To evaluate if slower migrating fragments are indeed larger fragments, we subjected end-labeled, chondroitinase treated chondroitin sulfate to gel filtration chromatography (Supplementary Information Fig. 2). Size separation matched the separation observed after gel electrophoresis, except for the smallest fragment observed. This fragment likely represents the linkage region, which due to the relative low amount of negative charges, migrated close to band 2 (see also Fig. 2c, lane 3 (60′), in which a novel band appeared (arrow) after prolonged enzyme digestion.

### Bioavailablility of sequence-defined fragments

One major advantage of the method proposed here is the potential to interrogate the sequence-defined fragments by *e.g*. biomolecules like proteins, or by chemical treatment. The bioavailability of the blot-immobilized fragments was demonstrated by their susceptibility to *in situ* enzymatic digestion (Fig. 3e). The potential to apply chemical treatments was shown by the use of mercury(II) acetate, a chemical that will cleave off the terminal unsaturated uronic acid^17^ thus abolishing detection by antibody 2B6 (reactive with ∆UA-GalNAc4S), which can be restored by additional enzymatic treatment exposing new ∆UA-GalNAc4S groups (Fig. 3f).

### Comparison with sequence data from literature

The sequence deduced here is in line with the data from literature which indicates the chondroitin sulfate chain to be composed of 15 ± 3 disaccharides (7–8 kDa) of which ~ 5 disaccharides are 4-sulfated and clustered at the reducing end, the non-sulfated disaccharides being clustered at the non-reducing side^18^. It closely matches the sequence deduced by electrospray ionization Fourier transform-ion cyclotron resonance mass spectrometry (ESI FTICR-MS)^8^, including the transition zone with decreasing amounts of 4-O-sulfation. However, it differs in one main aspect: we found only one rather than two non-sulfated disaccharide at the reducing end. This may be due to incomplete enzymatic digestion, chondroitinases having a preference for scission at sulfated disaccharides. To overcome this, longer incubation times, combination of enzymes, or non-enzymatic fragmentation (*e.g*. by free radicals^19^ or nitrous acid in combination with hydrazinolysis^20^) may be probed. Another potential limitation is the possible anomalous migration pattern of small fragments with an irregular degree of sulfation. This may be counteracted by the use of negatively charged bioorthogonal probes, analogous to the fluorescent label aminonaphthale-trisulfonic acid^21^ used in PAGE, or by the application of gradient PAGE gels which separates primarily on the basis of size^22^.

### Sequencing complex glycosaminoglycans

In this paper we sequenced the chondroitin sulfate chain from the proteoglycan bikunin, one of the most simple glycosaminoglycans. Other proteoglycans harbor structurally more diverse glycosaminoglycans with major variability regarding the degree and type of O-sulfation, N-sulfation/acetylation, epimerization and chain length. Standard polyacrylamide gel electrophoresis allows the separation of glycosaminoglycan fragments up to 50–100 disaccharide in length and differing only one disaccharide for each other, allowing the full length chain to be sequenced^11^. Use of large polyacrylamide gels, applied in classical DNA sequencing, may be used for improved separation. A prerequisite for sequencing is the availability of homogenous preparations containing only one specific glycosaminoglycan chain. Purification of a specific proteoglycan from a selected tissue would be an initial step, and would be sufficient in case of proteoglycans harboring only one chain such as decorin, a dermatan sulfate proteoglycan associated with collagen fibrils. In case of multiple chains on a core protein -assuming the chains have different sequences- partial proteolysis may allow the generation of peptides with only one chain. Purification of homogenous glycosaminoglycan preparations may further be accomplished using various forms of chromatography, including immunoaffinity using antibodies specific for certain glycosaminoglycan domain structures, e.g. phage display derived antibodies^23^.

With regard to the sequence procedure a number of options are available. The most simple one would be to have antibodies available to all the possible terminal unsaturated disaccharides, and visualize the sequence as has been shown in this study with the antibodies 2B6 and 1B5 (see Fig. 3a,b). However, only few such antibodies are currently available and an effort has to be made to generate more antibodies, e.g. by applying antibody phage display technology^24^. Alternatively, each blot-immobilized glycosaminoglycan fragment can be excised (see Fig. 3c) and individually analyzed for the chemical composition of its terminal saccharides using HPLC, MS or electrophoresis. To this extent a number of approaches are available. For instance treatment of individual blot-immobilized fragments with lyases such as chondroitinases or heparinases will reveal new fragments which can be analyzed biochemically. By comparison with neighboring bands the nature of the terminal disaccharide can be deduced. Alternatively, the unsaturated terminal uronic acid residue can be cleaved off by mercury acetate (Fig. 3f) resulting in the exposure of the aminosugar (GalN/GlcN) as the terminal monosaccharide. Next, the sequence can be probed by a set of exoenzymes specifically removing certain chemical moieties from the non-reducing end of the chain (using e.g. GalNAc-4-sulfatase, GalNAc-6-sulfatase, N-sulfatase, glucuronate-2 sulfatase, iduronate-2 sulfatase, glucuronidase, iduronidase, hexoaminidase). Virtually all exoenzymes involved in the degradation of glycosaminoglycans are known and many of them are commercially available. Susceptibility to these enzymes can be evaluated using PAGE^4^. Instead of mercury acetate, bacterial unsaturated glucuronyl hydrolases may be used which specifically remove unsaturated uronic acid residues, thus exposing aminosugars^25^. Finally, the terminal unsaturated disaccharide can be reduced using the oxymercuration-demercuration reaction^26^. When blot-immobilized fragments are subsequently treated with lyases, only one saturated disaccharide will be liberated amidst a number unsaturated ones, and this can be characterized by HPLC and MS technology^26,27^.

In conclusion, we report a methodology to sequence glycosaminoglycans based on covalent immobilization and identification of *only* those glycosaminoglycan fragments containing the original reducing end of the full-length molecules. Fragments are bioavailable allowing the study of sequence-defined interactions with biomolecules such as proteins, but also cells. The method uses commercially available chemicals and standard laboratory equipment and may spark studies to the role of glycan sequences in health and disease.

## Materials and Methods

### Materials

Bikunin was from Aviva Systems Biology (San Diego, CA); chondroitin sulfate A, 4-azidoaniline HCl, NaCNBH_3_, chondroitinase ABC from *Proteus vulgaris*, hyaluronidase type IV-S from bovine testes, β-glucuronidase from bovine liver type B-10, acrylamide/bis-acrylamide, TEMED, MES (morpholino-ethane-sulfonic acid) monohydrate, mercury(II) acetate, AMAC (aminoacridone), EDC (N-(3-dimethylaminopropyl)-N′-ethylcarbodiimide), NHS (N-hydroxysuccimide) and acetonitrile from Sigma-Aldrich (St Louis, MO); chondroitinase AC from IBEX, Montreal, Quebec, Canada; anti-chondroitin sulfate (unsaturated) antibody 1B5 and 2B6 from MDBioproducts (St Paul, MN), goat polyclonal anti-mouse IgG IRDye680RD®, goat polyclonal anti-mouse IgG IRDye800CW® and DBCO-IRDye680RD® from Li-cor Biosciences (Bad Homburg vor der Höhe, Germany); BCN (bicyclononyne)-PEG_3_-amine from Synaffix (Oss, The Netherlands); dibenzylcyclooctyne-amine (DBCO), trans-cyclooctyne (TCO)-amine and DNP-azide (6-(2,4-dinitro-phenylamino)-hexanoic acid (3-azido-propyl)amide) from Jena Bioscience (Jena, Germany); Cy7-methyltetrazine and tetrazine-amine from Click Chemistry Tools (Scottsdale, AZ); TCO-PEG_3_-amine and tetrazine-oxyamine HCl from Conjuprobe (San Diego, CA), azido-PEG_3_-oxyamine from Uptima Interchim, Montlucon Cedex, France; BiodyneC® transfer membrane from Pall Life Sciences (Pensacola, FL); Hybond-n (neutral) nylon transfer membrane from GE Healthcare Life Sciences (Piscataway, NJ), unsaturated chondroitin sulfate di-, hexa- and decasaccharides from Iduron (Cheshire, United Kingdom); Bio-Gel® P-6 media fine, 45–90 µm (wet) from Bio-Rad (Hercules, CA) and crimp vials from Chromacol (Herts, United Kingdom). All other chemicals used were purchased from Merck (Darmstadt, Germany). Chondroitinase AC-II from *Arthrobacter aurescens* was a generous gift from Prof. K. Sugahara, Faculty of Advanced Life Sciences, Hokkaido University, Japan. Antibody 1B5 was a generous gift from Profs. B. Caterson and C. Hughes (School of Biosciences, Cardiff University, United Kingdom).

All experiments were performed at ambient temperature (22 °C), unless stated otherwise.

## Methods

### End-labeling of full-length chondroitin sulfate and chondroitin sulfate saccharides with bioorthogonal probes

Full-length chondroitin sulfate was released from the proteoglycan bikunin by alkaline β-elimination creating a reducing end. To a bikunin solution (10 mg/ml in MilliQ water of which ~1/3 part is chondroitin sulfate^18^), an equal volume of 1 M NaOH was added followed by incubation overnight at 4 °C. The mixture was neutralized to pH 7 using 5 M HCl and lyophilized. Labeling was performed by adding twice the initial volume of the bikunin solution using 0.1 M BCN-PEG_3_-amine or 4-azidoaniline in 100 mM NaAc/150 mM NaCl, pH 5.4 followed by incubation for 15 min. Reduction was performed by adding an equal volume of 1 M NaCNBH_3_ in MilliQ water followed by incubating for 4½ h at 45 °C. Glycosaminoglycans were precipitated overnight using 5 volumes of ice cold 100% methanol at −20 °C and centrifuged for 60 min at 18,000 × *g*, 4 °C. The resulting supernatant was removed and the pellet dried in a desiccator.

Labeling of chondroitin sulfate saccharides was performed similarly, but without the alkaline β-elimination procedure and precipitation step. The probes used for labeling are listed in Table 1.

### Enzymatic fragmentation

#### Digestion with chondroitinase AC/ABC

Chondroitin sulfate-containing pellets were dissolved in chondroitinase AC-II digestion buffer (50 mM NaAc, pH 6.0) or chondroitinase ABC/AC digestion buffer (25 mM Tris-HCl, 2 mM MgAc_2_, pH 8.0) at a concentration corresponding to 1–10 mg/ml of the original bikunin preparation, and 0.01–0.05 units chondroitinase AC-II/ml or 0.5 units chondroitinase ABC/ml were added. In one occasion a combination of ABC and AC was used both at a concentration of 10 mU/ml. Incubation was at 37 °C and digestion was stopped by boiling for 3 min followed by centrifugation for 3 min at 18,000 × *g*, 4 °C. The supernatant was evaporated using a Savant Speed Vac concentrator and samples were stored in 25% glycerol at −20 °C.

#### Digestion with hyaluronidase/glucuronidase

Chondroitin sulfate-containing pellets were dissolved in hyaluronidase digestion buffer (20 mM NaCl/30 mM Na-phosphate buffer, pH 5.3) at a concentration corresponding to 1.5 mg/ml of the original bikunin preparation, and digested with hyaluronidase type IV-S (700 units/ml). Samples were incubated as described above and split into two parts, one additionally treated with β-glucuronidase type B-10 (700 units/ml) in digestion buffer (100 mM NaAc, pH 3.5). Samples were incubated for 3 h at 37 °C and handled as described above.

### Gel electrophoresis and selective staining of labeled fragments

A 30% polyacrylamide gel for a Bio-Rad Mini-Protean® system consisting of 3 ml 40% acrylamide/bis-acrylamide, 1 ml 1.5 M Tris-HCl, pH 8.8, 20 µl 10% APS (ammonium persulfate) and 2 µl TEMED, and an 8% stacking gel of 250 µl 40% acrylamide/bis-acrylamide, 310 µl 0.5 M Tris-HCl, pH 6.8, 673 µl MilliQ water, 10 µl APS and 1 µl TEMED was prepared. As running buffer 25 mM Tris-HCl/0.2 M glycine was used. After pre-running the gel for 30 min at 70 V, samples were run at 200 V. The amount of chondroitin sulfate loaded was calculated on the basis of the original amount of bikunin used at the start of the experiment, 1/3 of this being chondroitin sulfate^18^. In general, this corresponded to 5–50 µg/lane. The gel was incubated in 0.1% Alcian Blue in 10% HAc, washed in 10% HAc, washed with MilliQ water and subsequently stained overnight with the respective reaction partner conjugated to an IR-dye (1 µg/ml in MilliQ water, *e.g*. DBCO-IRDye680RD^®^ for azidoaniline labeled glycosaminoglycans and Cy7-methyltetrazine for BCN-PEG_3_-amine labeled glycosaminoglycans). The gel was washed for several days with MilliQ water. Image processing was by Odyssey CLx Imaging System and Image Studio Lite, version 5.0 software and Microsoft Powerpoint/Adobe Photoshop software. After visualization, the gel was silver stained by incubation for 15 min in 50 ml 5% sodium bicarbonate in MilliQ water followed by staining in a mixture of 30 ml 5% sodium bicarbonate and 30 ml 0.2% silver nitrate/1% tungstosilicic acid/0.2% ammonium nitrate. The reaction was stopped by incubation of the gel in 30 ml 10% HAc.

### Functionalization of blot paper with bioorthogonal probes

BiodyneC® membranes (blot paper, 9 × 7 cm, 0.45 µm) were rinsed in 50 mM MES buffer, pH 5.0 for 30 min followed by incubation with 3 ml 2 mM DBCO-amine or 0.5 mM tetrazine-amine in 33 mM EDC/6 mM NHS in 50 mM MES buffer for 4 h on a roller bank. The reaction was stopped by replacing the solution with 0.1 M Na_2_HPO_4_, incubation for 30 min followed by wash steps for 30 min with 1 M NaCl, 2 M NaCl and 6 times with MilliQ water, respectively. Membranes were air dried and stored in the dark.

### Blotting and immunochemical detection

4-Azidoaniline labeled chondroitin sulfate was transferred from the gel to DBCO amine-functionalized blot paper by capillary action using phosphate buffered saline (PBS, pH 7.3) overnight. BCN-PEG_3_-amine labeled chondroitin sulfate was transferred from the gel to a tetrazine-amine functionalized blot paper by electroblotting for 30 min at 100 V in 25 mM Tris-HCl/0.2 M glycine containing 20% methanol. After blotting, membranes were air dried for at least 6 h in the dark.

For immunochemical detection, membranes were incubated for 2 h in blocking buffer (2% bovine serum albumin in PBS containing 0.1% Tween-20 (PBST)) and incubated with antibodies recognizing unsaturated terminal disaccharides. For ∆UA-GalNAc, antibody 1B5 (1:50 dilution in blocking buffer, overnight incubation) was used; for ∆UA-GalNAc4S, antibody 2B6 (1:100 dilution in blocking buffer, 1 h incubation) was used. Membranes were washed 3 times for 10 min with PBST followed by 1 h incubation with goat polyclonal anti-mouse IRDye680RD® or goat polyclonal anti-mouse IRDye800CW® (both 0.1 µg/ml in blocking buffer) as secondary antibody. Membranes were washed again and image processing was as described.

### Detection of the first disaccharide bound to linkage region by RP-HPLC

Creation of a reducing end, labeling with BCN-PEG3-amine, digestion with chondroitinase ABC, separation on a 30% polyacrylamide gel, transfer to tetrazine-amine functionalized membrane and staining with antibody 2B6 was as described.

To analyze the terminal disaccharide, a part of the unstained blot paper corresponding to the position of the 2B6 positive stained band was cut out and incubated overnight at 37 °C with chondroitinase AC-II (5 mU in 0.5 ml 50 mM NaAc, pH 6.0). The sample was centrifuged for 3 min at 18,000 × *g* and the supernatant was transferred to a new vial. Digestion was stopped by boiling for 3 min followed by centrifugation for 3 min at 18,000x*g* at 4 °C and samples were lyophilized. As reference disaccharides 10 µl of a 0.1 M mixture in MilliQ water of 8 different unsaturated chondroitin sulfate disaccharides was also lyophilized.

Samples were labeled with 5 µl 0.1 M AMAC in DMSO/HAc (17:3) and 5 µl 1 M NaCNBH_3_ with conditions as described above. After addition of 190 µl 50% DMSO, the samples were centrifuged for 3 min at 18,000s × *g* and supernatants were transferred to 300 µl crimp vials and stored at −80 °C.

The samples were analyzed by reversed phase HPLC with a gradient elution (adapted from^28^) on a Shimadzu Prominence HPLC system with a Waters X-Bridge Shield column (100 × 4.6 mm; 3.5 µm). The column was equilibrated with 98% buffer (60 mM NH_4_Ac, pH 5.6) and 2% acetonitrile. The following program was used for the separation of unsaturated disaccharides: 1 min at equilibration conditions, a linear gradient step in 2 min to 4% acetonitrile, a separation gradient step from 4 to 15% acetonitrile in 23 min followed by a wash step with 60% acetonitrile for 5 min and back to equilibration condition for 9 min. The flow rate was 1.5 ml/min and temperature 25 °C. Fluorescence detection was performed using excitation and emission wavelengths of 442 nm and 520 nm, respectively.

### Size separation of chondroitin sulfate saccharides by FPLC

Creation of a reducing end, labeling with 4-azidoaniline, digestion with chondroitinase ABC and AC-II was performed as described. Samples were analyzed on a ÄKTA purifier GE Healthcare Life Sciences) with a Bio-Gel® P-6 column (65 cm × 1.6 cm; 45–90 µm) after equilibration with 1 bed volume of 0.25 M (NH_4_)_2_CO_3_ at a flow rate of 0.08 ml/min. Two ml samples were collected and 200-fold concentrated using a Savant Speed Vac concentrator. Samples were applied on 30% polyacrylamide gel and stained with DBCO-IRDye680RD^®^ as described.

### Removal of unsaturated uronic acid residues using mercury(II) acetate

CS decasaccharide and chondroitinase ABC digested chondroitin sulfate A were labeled with azidoaniline and spotted on DBCO-functionalized transfer membranes. After 30 min, the membranes were washed 3 times with MilliQ water for 10 min followed by 90 min incubation with 17.5 mM Hg(OAc)_2_ in 50 mM NaAc, pH 5.0 at 37 °C^17^. Membranes were washed 3 × 20 min and overnight with MilliQ water and stained with antibody 2B6 and goat polyclonal anti-mouse IRDye800CW® and analyzed as described above. Membranes were additionally treated overnight with chondroitinase ABC (5 mU in 1 ml), stained again with 2B6 antibody and IRDye800CW® and visualized as described.

## Electronic supplementary material

Supplementary Figures
