# Supplementary material for: Sequencing of glycosaminoglycans with potential to interrogate sequence-specific interactions
Source: Sci Rep. 2017 Nov 1;7:14785. doi: 10.1038/s41598-017-15009-0 (PMC5665995; doi:10.1038/s41598-017-15009-0)

Supplementary Information

Sequencing of glycosaminoglycans with potential to interrogate sequence-specific interactions

Toin H. van Kuppevelt[1], Arie Oosterhof, Elly M.M. Versteeg[1], Emina Podhumljak[1], Els M.A. van de Westerlo[1], Willeke F. Daamen[1]

[1]Department of Biochemistry, Radboud Institute for Molecular Life Sciences, Radboud university medical center, PO Box 9101, 6500 HB Nijmegen, The Netherlands

Supplementary Fig. 1.

General characteristics of the bikunin preparation used.

a. SDS-PAGE of bikunin (right lane) showing an apparent molecular weight of about 32 kDa (Coomassie Blue staining). Left lane: molecular weight markers.

b. PAGE of bikunin before (left lane) and after (right lane) alkaline ß-elimination (Alcian Blue/silver staining).

c. RP-HPLC disaccharide analysis of chondroitin sulfate derived from bikunin (chondroitinase ABC digestion, AMAC-labeling, lower panel). Note the presence of ΔUA-GalNAc and ΔUA-GalNAc4S disaccharides. Reference disaccharides are given in the upper panel.

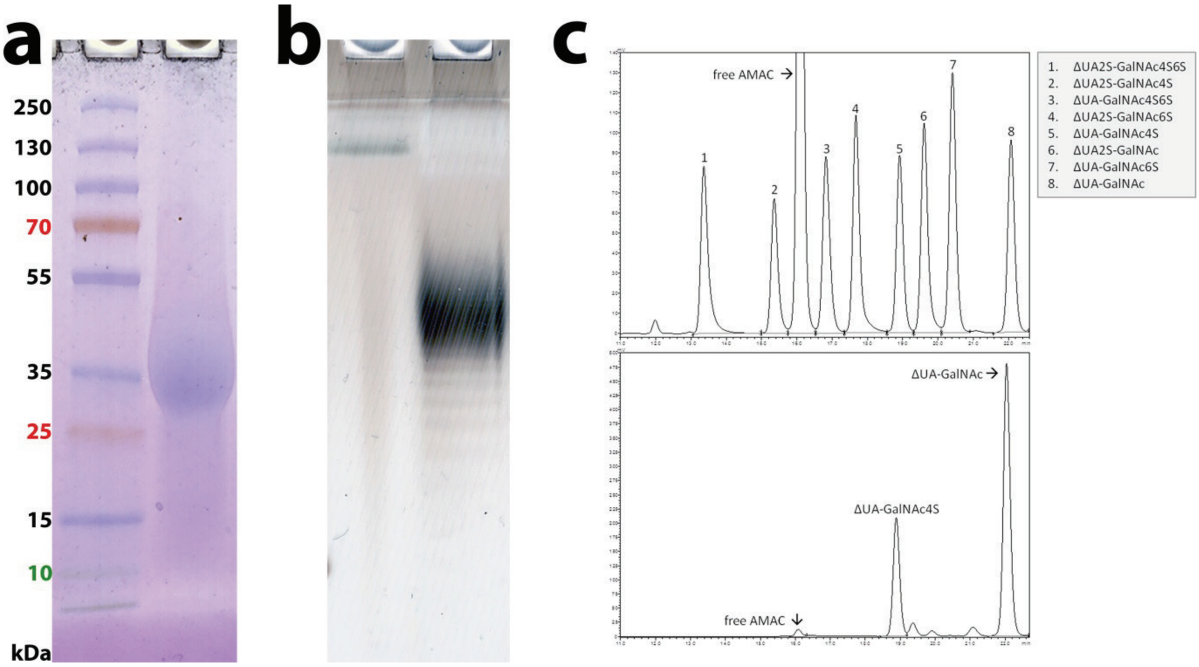

Supplementary Fig. 2.

Gel filtration of end-labeled, fragmented chondroitin sulfate derived from bikunin.

Chondroitin sulfate (~260 µg) derived from bikunin was end-labeled with azidoaniline, treated with chondroitinase AC-II (20 and 60 min combined), and fractionated by FPLC using a Biogel P-6 column. Fractions (2 ml) were collected, lyophilized, subjected to 30% polyacrylamide gel electrophoresis and visualized by incubation with DBCO-IR Dye680RD®. Note that the separation on PAGE is in accordance with the separation on gel filtration column, except for the smallest fragment (arrow) which co-migrates with the fragment obtained by exhaustive digestion with chondroitinase AC-II (lane labeled "60' ACII-ase"), and likely represents the linker region. A selected number of fractions are shown. Note that the fractions A8-B6 and B11-D1 are of two separate gels.

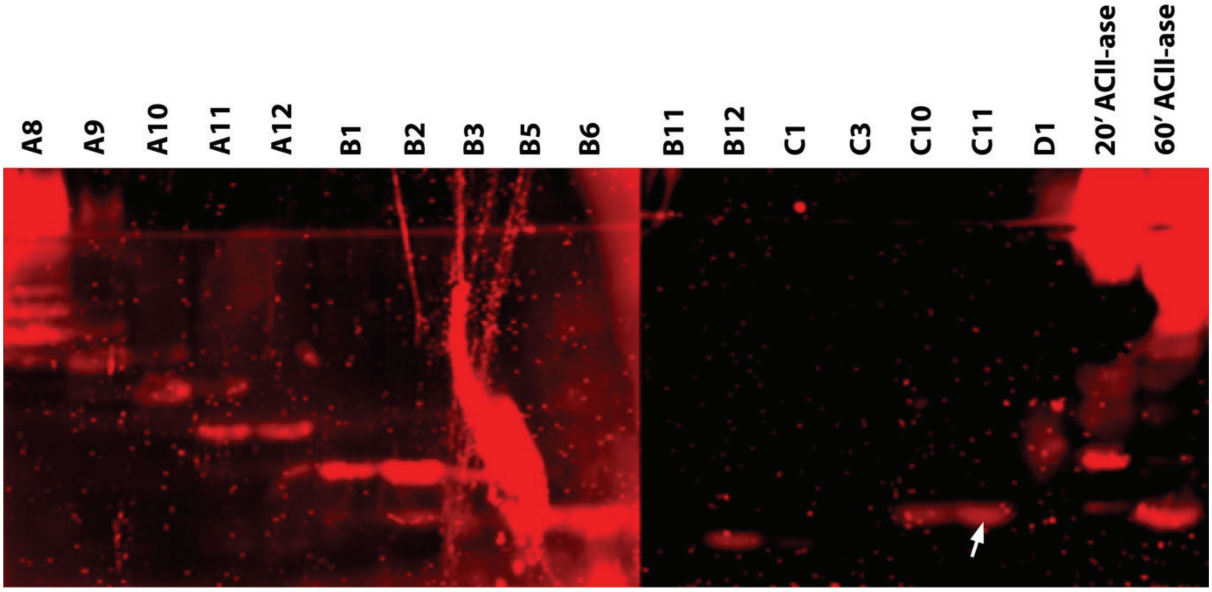

Supplement: Supplementary file 1 — Supplementary Figures [file 41598_2017_15009_MOESM1_ESM.pdf]
